# Disparate emotions? The play of emotions in clinical histories and patients’ letters (Pinel Sanatorium, SP/Brazil – 1929–1944)

**DOI:** 10.1017/mdh.2025.10037

**Published:** 2026-04

**Authors:** Yonissa Marmitt Wadi

**Affiliations:** State University of Western Paraná (UNIOESTE); National Council for Scientific and Technological Development (CNPq), Brazil

**Keywords:** emotions, letters from patients, social markers of difference, madness, medical records, Pinel Sanatorium

## Abstract

The Pinel Sanatorium, the brainchild of Doctor Antonio Carlos Pacheco e Silva, a leading figure in Brazilian psychiatry, was inaugurated in 1929 in São Paulo as a private institution. It operated until 1944, during which time it recorded approximately 4,500 hospitalisations. In 30 psychiatric records, in addition to the usual clinical records, such as the Psychiatric Examination – in which the doctor records the elements he deems essential for identifying the mental illness from different sources of information, such as those provided by family members – attachments were found containing letters and short texts written by the inpatients. Addressed to different people, these letters, which were retained and evaluated by the doctors, played a central role in assessing the psychiatric conditions of the inmates. However, by being considered historical sources that reveal the ‘point of view’ of the mad, these documents are fundamental to the development of innovative approaches in the field of the history of madness and psychiatry. Based on the articulation between the context in which these records were produced, the social markers of difference that constitute the subjects, as well as the emotions expressed by the people who wrote them, the article sets out to answer two questions: (1) How the emotions expressed – both by the inmates and by their loved ones – were interpreted by psychiatrists and used to formulate diagnoses, and to define treatments and prognoses; (2) What meanings these emotions took on for the inmates themselves, in other words, how they put their experiences and subjectivities on display.

## Introduction

Natanael was a teacher and the owner of a school, but he was also a homosexual.^1^ Dolores was a housewife and mother of three, but she read literature on women’s rights and wanted to sing tangos on a radio programme. Marília was also a housewife and mother of two, but she had left her husband to live in the capital with an older man, whom she ended up killing. Luísa, also named as a housewife, and Lúcio, a shopkeeper, became involved – in different ways – in an armed conflict that raged in São Paulo in 1932. Manuel, a well-known pharmacist in his town, shot the local parish priest because he believed he had seduced his wife. Valentina, another woman mentioned as a housewife in her psychiatric records, but noted as a society lady, no longer wished to live with her violent husband, who, in reaction, requested her civil ban.^2^ All the people mentioned were interned at the Pinel Sanatorium between 1929 and 1944.

These people were identified by various social markers of difference, in addition to the ones I have highlighted when briefly describing them. These classificatory categories (gender, sexuality, occupation/profession, social class, age, race, ethnicity, etc.), ‘understood as local, historical and cultural constructions’^3^ and articulated in a specific or generic way for each subject, show how differences ‘can contain implications in terms of hierarchy, asymmetry, discrimination and inequality’.^4^ In this sense, emotion is another important marker in classifying these bodies. Emotion is found ‘in words, experiences, virtues and vices’, which ‘often appear in groups and sequences’^5^ as we read, for example, in the psychiatric report about Lúcio – ‘nervous, impressionable; with an unstable mood, he went from joy to sadness for no justifiable reason’^6^ – or in the letter written by Luísa, attached to her medical record – ‘I realised, however, that I could not (…) suffer in silence, that my dignity could not withstand these affronts, that my love as a woman could not withstand the shock of return and would succumb.’^7^

Around 4,500 people were admitted to the Pinel Sanatorium between the time it was founded in 1929 as a private hospital and the time it was bought by the São Paulo state government in 1944, which transformed it into a public hospital.^8^ Natanael, Marília, Luísa, Lúcio, Dolores, Manuel, and Valentina are part of a group of just 30 people whose writings have been located in their psychiatric records.^9^ These writings, as was usual in many psychiatric institutions, were retained in their psychiatric records as documents that could contribute, through the description of acts, behaviours, disorders, disturbances, suffering, emotions, etc., in the medical assessment of what was happening to them and, above all, in assigning a diagnosis.^10^

The Pinel Sanatorium was the brainchild of Dr Antonio Carlos Pacheco e Silva – an exponent of psychiatry in São Paulo and Brazil at the time – and its propaganda attested to the fact that it followed the most advanced precepts of medical science, with innovative treatments.^11^ Aimed at wealthier social groups – members of the agrarian, commercial, or industrial elites – and segments of the middle classes who could afford the cost of hospitalisation, the institution was built on the outskirts of the state capital of São Paulo, in the area of the current district of Pirituba. Inspired by North American hospitals, it had 18 pavilions.^12^

The very name ‘sanatorium’, instead of psychiatric hospital, already sought to differentiate that place from other similar ones aimed at the hospitalisation, treatment and, perhaps, cure – within what was understood as this at that time and place – of mad people. The latter name, even in those days, referred to overcrowding, ineffective treatment and a lack of many things – staff (mainly doctors and nurses), modern instruments to apply modern therapies, etc. – while the former referred to a social imaginary of sanatoriums as places away from the hustle and bustle of the cities, quiet, wooded, where someone could escape from the hardships of everyday life, through rest, the peace provided by immersion in nature, and still be treated with the most modern therapies, by well-trained doctors and other specialised staff, as the institutional propaganda would have you believe.^13^

As the term ‘private’ indicates, the benefits of a sanatorium of this type were certainly not available to everyone, but only to those who could afford the cost of hospitalisation. At Pinel, the daily rates covered accommodation, food, nursing services, and medical care, but they did not include possible treatments – such as insulin therapy, malaria therapy, and Cardiazol therapy – tests, such as urine tests or the Wassermann reaction, or even laundry, all of which were charged for separately.^14^

Many studies have been carried out on the Pinel Sanatorium, its daily life, therapeutic practices, its founder, and its inmates from different perspectives situated in the historiographical field. Many works focus on the scientific and political career of the psychiatrist Antonio Carlos Pacheco e Silva, taking Pinel as one of the loci of his action and contrasting his practices with his work as director of the Juquery Hospital.^15^

A significant portion of the studies focus on analysing the women admitted to the sanatorium, problematising, from their psychiatric records, above all, the medical discourse about them and related issues. Among these issues, the ‘socio-normative aspects that characterised the female gender and that were defended as criteria of normality’ stand out, as well as the ‘reasons’ that justified the exclusion of some women from social life, classifying them as ‘deviant’. These studies look at the specificities of this classification and its relationship with themes such as ‘morality, sexuality, desires for freedom, taste for work, among others’.^16^ The significant research on this theme is undoubtedly related to the growth of studies on the history of women and gender relations in Brazilian universities, especially in the 1990s and 2000s. In addition, part of this interest is based on an initial historiographical confusion: the mistaken idea that the Pinel Sanatorium was an institution exclusively for women, which only became a reality after being sold to the state government in 1944.^17^

Some works focus on groups of inmates at the Pinel Sanatorium, based on social markers of difference such as age, generation, nationality, or ethnicity;^18^ in others, the central objective is to discuss the mental hygiene and eugenics movement, whose main herald in São Paulo was the psychiatrist Pacheco e Silva.^19^ Others use the sources produced at the hospital, especially the medical records, to discuss issues relating to the social history of language.^20^

The scholarship cited above, along with other related works, made a specific and significant contribution to the field of the history of madness and psychiatry, as well as enriching more general studies on the history of medicine. This contribution was made by taking as its object of study an institution built at a time when the hygiene movement, eugenics, and biological therapies were spreading, in a context in which psychiatry was undergoing profound transformations. In addition, the research highlighted the role of a doctor who became an internationally recognised psychiatric authority and the importance of the city where the institution was located, which would become the first Brazilian metropolis. In this way, these studies have deepened our understanding of the institutional dynamics, medical practices, and social relations that marked psychiatric activity in that period.

Despite incorporating sources such as psychiatric records, studies on the Pinel Sanatorium have hitherto had a fundamental shortcoming: they have not shifted the place of enunciation by neglecting the narratives of the inmates: those that express what Roy Porter called the ‘patient’s point of view’.^21^ These narratives are mostly only accessible through the filter of medical reports and, more rarely, appear in direct records, such as handwritten texts, recorded speeches, or artistic expressions, including drawings and paintings on the walls of the institutions. Although some works mention specific excerpts from these narratives, they tend to favour discussion from a medical or institutional perspective. Even when they problematise this perspective – criticising its excesses, the model of care and its limitations – they end up silencing the subjective experience of people considered mad, their perceptions of what they experienced, their emotions, and their forms of expression.

In recent decades, historiographical studies on madness and psychiatry have consolidated the narratives of people considered mad as legitimate and reliable sources.^22^ Using these sources has enabled innovative interpretative approaches within the field^23^ and offered new perspectives for understanding madness and psychiatric intervention practices in general medicine and psychiatry itself.^24^

However, studies on the Pinel Sanatorium still lack an analysis that considers the inmates’ ‘point of view’ as a central perspective in constructing the institution’s history. By shifting the place of enunciation, an analytical and interpretative turn becomes possible, broadening the understanding of this history. In this work, I follow the approach inaugurated by Roy Porter, which I have explored for some time. I argue that problematising the ‘patient’s point of view’ contributes to a more refined understanding of the relationships established in the microcosm of a psychiatric institution, as well as the broader socio-cultural context that determines which individuals become susceptible to psychiatric hospitalisation.

Incorporating the narratives of people considered mad as sources and objects of analysis makes it possible to highlight both the contrasts and the similarities between those who have the authority to judge the sanity of others (such as doctors and family members) and those who occupy the position of patients. It also makes it possible to understand the effects of medical practices, the forms of resistance to them, and the patients’ subjectivity beyond their instrumentalisation in medical expertise.

Another possibility this type of source opens up is to discuss how the emotional device operates within the institution. This is the ‘set of discursive strategies – scientific, religious, legal and moral, institutional and aesthetic — that each historical era constructs and collaborates to generate, obtaining a different emotional capital for each sex’.^25^ Similarly, it is possible to analyse the emotional resistance to norms, as well as the negotiations between the mad, their doctors, and families, broadening the understanding of subjective experiences in the psychiatric context.^26^

Studies that analyse emotions highlight their fundamental role in the medical understanding of various illnesses, including mental illnesses. According to Alberti, historians are growing aware that medical diagnoses are culturally situated. Thus, the emergence of specific clinically recognised emotional pathologies – such as hysteria, hypochondria and neurasthenia – should not only be seen as the result of changes in emotional experience, but as a social, political and cultural construction, reflecting, above all, gender and class dynamics. As the author points out, the extent to which emotions can and should be controlled, recognised, denied, or revealed has been central to debates about health and illness from the Middle Ages to the present day. These debates, far from being merely theoretical, have been incorporated into everyday life, influencing the structure of social relations and the production of medical knowledge.^27^

In the light of these considerations, the first objective of this article was to analyse how the emotions expressed by inpatients, as well as those reported by people with emotional ties to them, recorded both in psychiatric records (such as the Psychiatric Examination) and inpatients’ manuscripts, were assimilated by psychiatrists and used to formulate diagnoses, define treatments, and predict prognoses. In addition, I seek to understand how the social markers of difference were articulated to the emotional device in force in that historical context, influencing the construction of the medical perception of these emotions.

The second objective of this article was firstly to identify which emotions related to these people’s experiences were recorded in their manuscripts and then try to understand the meanings that these emotions took on for them – in other words, how they highlighted their experiences and subjectivities and functioned as tactics of resistance and negotiation.

I understand emotions as historically situated events, integrated into culture, social interactions, and intersubjective processes over time, and not limited only to the psycho-physiological dimension of the human being.^28^ Emotional expressions – like bodily expressions – are signs of illness whose manifestation varies according to context, content, and form. Socially constructed and culturally regulated, these expressions fulfil a social function by guiding human action and acting as elements of identity and belonging to a group.^29^

From this perspective, I look for expressions and terms in the letters analysed that reveal the emotions experienced in and through the experience of hospitalisation. Although their meanings change over time, these words are expressed and understood in particular ways in the specific context studied. It is fundamental to analyse emotions within the context in which they circulate and produce effects since they are intrinsically related to the values, objectives, and situations that subjects mobilise to make sense of the world and themselves.^30^

Finally, it is noteworthy that the 30 psychiatric records from the Pinel Sanatorium containing manuscripts produced by inmates represent a small fraction of the approximately 4,500 admissions recorded during the period. These records also relate to a specific segment of the population that commonly occupies psychiatric hospitals, which therefore does not allow for generalisations about all inmates in institutions of this type. However, they constitute unique sources, whose relevance lies in providing access to the experiences and subjectivities of subjects subjected to the psychiatric regime. The choice of a restricted documentary corpus is supported by widely consolidated historiographical references, which value the thorough analysis of scarce or fragmentary sources, especially when they have high heuristic potential, as is the case with the documents in question. Both nationally and internationally,^31^ the literature emphasises that, given the rarity of this type of material, the exhaustive analysis of available records is not only methodologically legitimate but also indispensable. In this study, such an approach allows us to reconstruct individual experiences, infer collective constructions, shed light on institutional practices, decipher medical and social discourses on madness, and recognise the tactics of resistance and negotiation of inmates in the face of institutional norms, by articulating social markers of difference (such as class, gender, and sexuality) with the emotional apparatus prevailing in that society during that historical period. Thus, the results presented should be understood in the light of these limitations, prioritising, however, the qualitative and interpretive potential of the testimonies for understanding experiences and practices in the context of private mental health care during the period analysed.

Given the above, the article is divided into two sections, besides this presentation and the final considerations. In the first section, I problematise the information about the people who wrote letters and other texts, contained in clinical histories written by psychiatrists. In these sources, which allow us to get to know the daily life of the institution as a whole and the particular issues related to each inpatient, I try to address the first objective that makes up the problematic of this article, that is, how the emotions of the inpatients were read and operationalised by the medical group, in the composition of a diagnosis, in the indication of treatments and in the prognostic perception of what was happening. In the second section, I try to answer the second objective by reflecting on the emotions related to the experiences (past and present) of the inpatients, expressed in the movement of their writing, and how these somehow constructed these subjects. Articulating the social markers of difference with emotions is transversal in the quest to elucidate the problem defined by the two objectives.

## The work of normal and abnormal emotions from a medical perspective

Various authors have problematised the use of psychiatric records, which contain patients’ clinical histories, as sources for historical and social studies. At the same time as they are understood as paradoxical narratives, ‘in a permanent tension between the poles of order and disorder’,^32^ these documents are considered reliable for investigating the daily life of institutions, within which, among other things, psychiatric practices are established that do not always coincide with the prevailing theories.^33^

Since Roy Porter’s influential work, they are also recognised as a meeting place between doctors and their patients,^34^ as well as sources that make it possible to access the latter’s experience.^35^ It cannot be ignored, however, that when the accounts of the inpatients are recorded in the psychiatric records – including those written in their hand, often retained for this reason – they end up being subordinated to the format imposed by these documents. They thus constitute ‘part of a narrative that aims to demonstrate, or not, the condition of madness’^36^ attributed to someone.

On the other hand, these true ‘discursive maps in which different voices converge’, as much as they are constituted as a hierarchical emotional device, where the voices of experts and family members have a higher status than the voices of the patients’,^37^ do not fail to make it possible – through the accounts of the life problems of those considered mad – to approach, rescue and broaden the understanding of ‘practices of emotional resistance of those who, on the map drawn by power, occupy the place of subalternity’.^38^

The psychiatric records at the Pinel Sanatorium consist of a few printed sheets (front and back) with fields to be filled in. The first sheet has spaces for identification data (name, age, ethnicity, marital status, profession, residence, origin), entry and exit dates, and the ‘History – Hereditary Background – Personal Background’ field. On the following pages, on both sides, there are spaces for information on the physical and mental examinations on entry (with date, time and ‘carers’ present); an indication of the ‘Persons authorised to visit the patient’; the ‘Somatic Examination’; the ‘Neurological Examination’; the ‘Psychiatric Examination’; and, finally, the ‘Diagnosis’ and ‘Course’.

Other printed sheets are present in most of them, such as prescriptions, control sheets for the application of biological therapies, and the questionnaire to be filled in by the carer at the time of hospitalisation. There are also some annexes such as laboratory tests (usually urine, faeces, blood, and the Wassermann reaction), telegrams from the institution’s directors or doctors to those in charge, letters and other writings from inmates that were not sent, as well as some letters received from family members.

All the documents that make up the corpus of psychiatric records – similar to those found in other Brazilian and foreign institutions in the same period – played a central role in the psychiatric assessment of the inmates. Among them, the questionnaire stands out, described as a complement to clinical observation, which, together with the others, provided evidence, clues, and signs considered fundamental to establishing the truth about each individual’s madness.

From the perspective of organicist psychiatry, the clinical observations made during hospitalisation, together with laboratory tests, should form the basis of the diagnosis, guide the treatments and determine the length of the patient’s stay, as well as the possibility of discharge, whether in the short or long term.^39^ An analysis of the psychiatric records, especially the entries under the heading ‘Psychiatric Examination’, reveals this was not the case. The medical reports often repeated information and opinions provided by the informants in the questionnaires, using them as indications of the existence of a psychiatric disorder or a mental illness.

The analysis of the Pinel Sanatorium is similar to the study carried out by López Sánchez and Gutiérrez Colín on the La Castañeda General Mental Hospital, one of Mexico’s leading psychiatric hospitals. According to the authors, the admission form and the medical interrogation were the two most relevant documents to assist doctors in deciding on the need for hospitalisation and formulating a diagnosis.

This interrogation aimed to gather information about the patient’s personal and family background, covering a wide range of data such as demographics, physical build, vaccination history, religion, schooling, and level of intelligence. It also sought to identify possible hereditary traits by analysing the family’s mental health conditions, as well as recording the patient’s previous illnesses. As the authors point out, knowing the medical history of the inmates allowed the doctors to identify ‘the characteristics of the illness, extravagances, sayings and acts considered irrational, as well as any behaviour considered abnormal’.^40^

Although the authors do not specify who provided all the requested information, it is possible to assume that it was the individuals being questioned themselves in many cases. As in the cases analysed in this study, the patients’ carers likely provided much of this information. Although the form at the La Castañeda General Mental Hospital contained a specific section for the details of the relative or person responsible for the hospitalisation – in which the reasons for the hospitalisation were questioned – the influence of these reports may have extended to other parts of the document. According to the authors, the information about events and behaviours provided by those who requested hospitalisation was considered fundamental for justifying the hospitalisation and, consequently, for formulating the psychiatric diagnosis.

In the context of both the La Castañeda General Asylum and the Pinel Sanatorium, psychiatrists built up their ‘diagnostic impression of the patient’ during interrogation, through questionnaires or clinical observation. In the process, they assumed a position of authority, consolidating themselves as the legitimate enunciators of the ‘truth’ about madness. Holders of medical knowledge, they were the ones who observed, interpreted, and decided, legitimising themselves as the only ones capable of unravelling the nature of mental illness and determining the fate of the inmates.^41^

With the information from the questionnaire and their personal observations, sometimes also considering the reports of the nurses who accompanied the patients daily, the Pinel psychiatrists constructed their opinions and established diagnoses about the inmates, whose writings were retained. In this case, one of the characteristic features is the oscillation of emotions, normal and abnormal, which I will illustrate with two stories of hospitalisation, that of Lúcio and Dolores.

On 14 September 1935, Lúcio was admitted to the Pinel Sanatorium, taken there by his father, Marino, due to repeated suicide attempts. In the Psychiatric Examination, the psychiatrist, based initially on the information provided by the father in the questionnaire, emphasised the presence of family members with ‘psychological disturbances’ and went on to describe Lucio’s emotional characteristics in detail. Since childhood, he was said to have been ‘always nervous, impressionable and in an unstable mood, going from joy to sadness for no justifiable reason’. There were also reports of an attempted suicide by ingesting four ‘cafi-aspirin’ tablets after a disagreement with his fiancée. After the wedding, Lúcio allegedly showed excessively jealous behaviour towards his wife, even hitting her on one occasion, although, according to the report, he immediately regretted that ‘motor impulse’. At the same time, he was described as a man who was ‘very upright in his business dealings’ and even showed ‘exaggerated scrupulousness’ in his commercial transactions.^42^

According to the doctor, Lúcio’s participation in the 1932 Revolution^43^ was a determining factor in the onset of his illness, which was already being announced. The triggering event was the bombing by an aeroplane of the ambulance he was driving. From this episode onwards, his nervousness would have increased considerably, accompanied by the appearance of ‘symptoms indicative of an impending psychosis’. It is reported that, on some nights, Lúcio would wake up startled, shouting: ‘There’s a bomb coming.’ Another significant event occurred months before his hospitalisation: the suicide of a close friend, who took his own life by shooting himself with a revolver. After this episode, Lúcio developed an obsession with the idea that he would suffer the same fate and even tried to take his own life.^44^

The doctor goes on to describe Lúcio’s various suicide attempts in the hospital, which he considers ‘inconsequential or even childish’. Among these attempts, he mentions episodes in which the patient plunged ‘his mouth and nose into a bowl of soup with the aim of drowning’ or tried to ‘asphyxiate himself in the water of the bathroom’. He then highlights Lúcio’s behaviour, which oscillated between ‘deep introspection’ and saying ‘disparate things’.

The diagnosis of schizophrenia, about which the doctor claims to have no doubts, is presented after a description of the patient’s specific characteristics. Lúcio showed ‘a tendency towards catatonic attitudes’, sometimes remaining ‘in an orthostatic position, erect, straight, staring at a point, without so much as a facial muscle twitching’. In addition, the doctor noted the presence of ‘auditory hallucinations’, reporting that Lúcio claimed ‘to be in daily communication with his loved ones’. The situation worsened, he went on, with the production of letters ‘without an addressee most of the time’, in which we can identify ‘cabalistic signs’, ‘reticence’, and a combination of ‘flagrant psychological breakdown, a marked tendency towards mysticism’ and ‘ideas of self-accusation’.^45^

After just over six months in hospital, Lúcio was discharged on 24 March 1936, showing a complete improvement in the symptoms of his illness. However, just twenty days later, he returned to the Pinel Sanatorium, presenting, according to the doctor’s report, ‘the entire symptomatological procession’ observed during his first hospitalisation. Subjected to insulin therapy, Lúcio showed ‘clear remissions’, albeit short-lived. As described by the doctor, ‘when the psychological disorders began, ideas of suicide set in’. This pattern characterised his second and final stay at the Pinel Sanatorium. On 28 November 1937, the doctor recorded the tragic outcome: ‘despite the vigilance to which he was subjected, he succeeded in his purpose, resorting to a strap’.^46^

Dolores was hospitalised at the Pinel Sanatorium for a period that coincided, in part, with Lúcio’s stay. Her first admission occurred on 2 January 1936 and lasted until the 16th. She was later readmitted on 25 January and remained at the sanatorium until 5 March of the same year. Although they did not live together directly, since the facilities for men and women were separate and physically distant, the relevant aspect for this analysis is how the doctors interpreted the emotions expressed by both. These interpretations brought them closer together, regardless of the significant differences in their profiles and life histories.

The Physical Examination carried out during hospitalisation and the subsequent Somatic Examination, which provided information on the patient’s biological aspects, such as physical constitution, quality of nutrition, blood pressure, and pulse, mentioned that she was a ‘married woman with four children’. Although irrelevant to Lúcio’s physical qualifications, this detail was considered an essential element in defining what was going on with Dolores, to the point of being included in a space not intended for this type of observation. In addition, it was recorded that Dolores had suffered an ‘induced abortion, lasting two months’ and that her ‘menstruation was scarce, but regular in time and duration’.^47^

As in Lúcio’s case, Dolores’ Psychiatric Examination also reported on her hereditary background. According to the doctor, the patient’s mother said that her father was ‘an inveterate alcoholic’ and mentioned that one of her brothers suffered from ‘encephalitis’. The rest of the doctor’s report is mainly based on information from her mother, a brother, and the sanatorium’s head nurse.

Initially, Dolores was described as ‘a young woman of 28, married for 11 years’, who ‘always lived very well with her husband’, with whom she had ‘four children, all alive and strong’. She was considered to have a ‘docile temperament’ and to be ‘somewhat withdrawn, totally dedicated to the household and looking after the children’, which changed around six months before her hospitalisation. From then on, according to her mother’s testimony as reported by the doctor, there was a drastic change in her behaviour, which alarmed the whole family. Dolores began to ‘show herself to be independent and wilful’, dedicating herself to reading ‘volumes and volumes on women’s rights, female emancipation etc.’ When her mother reproached her, she reacted with hostility, rejecting her advice and, at a certain point, abandoned the home, fleeing to Rio de Janeiro, where a brother later brought her. The doctor reports that both her husband and other family members agreed that the patient had undergone a significant transformation, manifested by an increase in vanity and an attempt to pursue an artistic career, an area for which she had never shown any aptitude or vocation.

In both the first and second hospitalisations, the Psychiatric Examination record, in inverted commas, statements are attributed to Dolores. In these reports, the patient – classified by the doctor as having ‘irreproachable behaviour’ – strongly reproached ‘the relatives who want to make her look sick’. She said that she ‘wasn’t meant to be a martyr’ and wanted to ‘get divorced’, justifying her attitude by the fact that her husband mistreated her and, in addition, she had feelings for another man. Dolores also declared that ‘no one could blame her feelings’ and regretted that ‘the country’s laws did not allow a full divorce, as she wanted, because she would not like concubinage’.^48^

She also told the head nurse that the abortion she’d had ‘just over a year ago’ had happened ‘because of her husband’s demands, who had always mistreated her, even physically’. However, her relatives’ ‘strict moral principles’ always put them on his side, so she was admitted to the sanatorium because her family preferred her to ‘be taken as mad, rather than less worthy’.^49^

When she was hospitalised for the second time, Dolores had spontaneously entered Pinel, motivated by the memory that there was a balance on her monthly payment and the desire to get away from her family. According to the psychiatrist, in the account in which he again puts Dolores’s sentences in inverted commas, her husband had discovered ‘a diary of hers in which he found proof’ of her betrayal, triggering a ‘violent scene’ that resulted in her expulsion from home. Dolores narrated the events from the beginning, emphasising the guilt of her husband, whom she accused of not preserving her affection and of taking her to a ‘bad step’, for which, however, she acknowledged ‘her culpability’. The patient stated that when she decided to leave home, she intended to do so definitively but was disappointed by the ‘attitude of the other’, who had refused to ‘keep her in his company’. This disappointment led to a suicide attempt by poisoning herself with lighting gas, from which she was saved and later hospitalised ‘on suspicion of madness’. In the end, Dolores reported that her brother, aware of the whole situation, had reproached her and advised her ‘to end her life’ as a way of ‘washing away her guilt’. ‘She therefore asked us for advice’, wrote the doctor.^50^

The psychiatrist does not mention what advice he gave Dolores, but he does indicate – in his following note some twenty days later – that ‘the patient’s behaviour continues to be very good’, but that ‘small occurrences, at first sight insignificant’, showed that the patient was not typical. From this, he concludes that her case looks like ‘another case of atypical degeneration’; in other words, this woman was in a border zone between normality and abnormality, as this diagnostic category advocated. In these cases, according to the medical theories in force at the time, the ‘degenerative basis is somewhat obscured by the predominance of the signs that are characteristic of them, (…) thus emphasising that there is no typical form.’^51^

This diagnosis contrasts with the one given to Dolores on her first admission, when she was in hospital for only fourteen days: ‘Incipient schizophrenia?’. The question mark, placed in freehand next to the typed diagnosis, was perhaps due to the difficulty in defining it, as it had only been a few days since she had been hospitalised. As Dolores had been ‘removed prematurely’, the uncertainty remained until the second hospitalisation brought, from the medical perspective, a little more certainty about what was wrong with her.^52^

In addition to the doubts raised by the psychiatrist, which seem frequent, as evidenced by the number of ‘undefined diagnoses’ and undiagnosed patients identified in my sample,^53^ even more profound questions arise. These include the following: what determined the conception of psychological abnormality and the need to hospitalise these two people?

Both were diagnosed with schizophrenia. In Lúcio’s case, the Psychiatric Examination reports relatively clear signs commonly attributed to this condition at the time, such as ‘a tendency towards catatonic attitudes, psychological disintegration, accentuated mysticism, ideas of self-accusation, alongside disorganised suicide attempts’ and auditory hallucinations.^54^ On the other hand, Dolores’ diagnosis of schizophrenia, which is initially questioned and later described as a possible atypical degeneration, is not characterised by the typical signs reported for Lúcio; instead, there are moral judgements regarding her behaviour, her desires and, above all, her relationship with her husband, elements which, according to the doctors, mark her psychological abnormality.

The description of the symptoms of Dolores’s illness is firmly anchored in a gender culture shared by family members and doctors. A clear example is the psychiatrist’s treatment of marital violence in the two cases analysed. Dolores’ complaints about abuse were dismissed by the family, especially by the mother, who said that ‘her son-in-law is a great husband’. The doctor adopted this view and attributed these complaints to the patient’s psychological abnormality. On the other hand, Lúcio’s aggression against his partner, justified in the questionnaire completed by his parent as being due to jealousy and a ‘motor impulse’ that he immediately regretted, was not considered by the doctor to be a central element of the ‘psychological breakdown’ presented by the patient.^55^

A common point of particular interest for this discussion is emotional oscillation as a marker of abnormality. Lúcio, who experienced the trauma of a conflict – in which he almost lost his life and witnessed countless deaths or situations bordering on death – showed intense emotions, described by his family members as intense traces of something present since childhood. Known for being nervous, impressionable, and unstable, oscillating between moments of joy and sadness, the post-conflict Lúcio began to manifest a constant state of alarm, often exclaiming ‘bombing is coming’ during nightmares. The sorrow and grief resulting from so many losses, including that of a close friend who had committed suicide, combined with the persistent fear of bombing even after it had stopped, seem to have led Lúcio to stop expressing any positive emotions, a condition considered typical of normality. Although the doctor identified these emotions, he did not analyse the traumatic impacts of the war – such as fear, hatred, and mourning – on the patient’s process of ‘psychological breakdown’.^56^ Finally, Lúcio’s suicide unequivocally confirmed, from a medical point of view, the abnormality of his emotional state.

From Pacheco e Silva’s perspective, as the ‘strong sex and, as such, more resistant to emotions’, a man could suffer the impacts of ‘psychogenetic factors’ on his ‘somatic sphere’, as happened with Lúcio, but ‘what about the woman who is, as we all recognise, of a fragile and delicate constitution, sensitive and vibrant’? The director of Pinel believed that ‘women’s lives’ gravitated ‘around apprehensions, worries and surprises, longings and disappointments, doubts and uncertainties, which accompany them throughout their lives’. Therefore, the ‘hyperemotional constitution, that state of vibratility so often observed in the female sex, predisposes women to a series of neuroses’.^57^

This clearly explained, from the medical perspective, what happened to the ‘docile’ and ‘withdrawn’ Dolores, a good wife and mother, a ‘devoted’ housewife, who had undergone a ‘radical change’, becoming ‘independent’, ‘wilful’, ‘hostile’, and ‘excessively vain’. She confessed to cheating on her husband, saying she ‘had no vocation to be a martyr’, and taking a stand against her family by claiming not to be mad. She had a hyperemotional constitution!

Dolores spoke of her sadness, which she said stemmed from the mistreatment she had received from a man she had married at seventeen ‘against her will’, with whom she had always been ‘incompatible’ and from an abortion caused ‘by her husband’s demands’, as well as the predictable tiredness of the perhaps heavy routine of domestic life and caring for four children. She also spoke of the hope of new possibilities in life; hope that arose from reading about ‘women’s rights’ and ‘women’s emancipation’. These readings may have shown Dolores that there was a world beyond the one where she lived, in which she could be a radio singer and love fully. However, none of this was possible considering the ideal of femininity that prevailed in her social group, despite the ongoing changes that challenged the boundaries between the sexes,^58^ as attested to by the publications she read.^59^

The gender patterns and roles established in Brazilian society, together with the prevailing emotional device – incorporated into both psychiatric theory and practice – meant that, in most cases, the objective criteria announced for the diagnostic assessment were replaced by ideal criteria, especially in the case of women and some men.^60^ By ceasing to be docile and withdrawn, probably sad, Dolores became abnormal – either a beginning schizophrenic or a proven atypical degenerate – and it remained for her, as a kind of resistance in accommodation,^61^ to tell the doctor that she had ‘good intentions for the future’, because she intended to ‘return to the company of her husband, taking into account the welfare of her little children’. This decision eventually allowed Dolores to be discharged permanently.^62^

## Playing with emotions

On 16 January 1935, Natanael Borges, a 25-year-old single man, teacher, and resident of the capital, was taken by ‘police inspectors’ to the Pinel Sanatorium at the request of his father, Lauro Borges. A little over four years later, on 17 April 1939, Manuel Feitosa Cardoso, a 47-year-old married man, pharmacist, and resident of Angatuba, in the interior of the state of São Paulo, was also taken to the Pinel Sanatorium, led by representatives of the police force and accompanied by several people from the locality where he lived.^63^

Although the police took both, the reasons for their admission to Pinel were very different. Manuel was taken to the sanatorium because he had shot the priest of the town where he lived since he believed that he had seduced his wife; Natanael, in turn, was taken because he ‘showed a tendency towards homosexual practices’. Manuel did not make it to his first month in hospital and was removed by his family, ‘against the advice’ of Pacheco e Silva himself, who signed the Psychiatric Examination, ‘as he was a persecuted-persecutor of maximum potential danger’; Natanael stayed in hospital for around six months and was discharged, as indicated by the report in his psychiatric records: ‘Apart from a certain apathy and indolence, we noticed nothing else during Mr Natanael’s stay at the Sanatorium, who said he regretted his behaviour and was ready to regenerate after returning to ordinary life.’^64^

Without any diagnosis, Natanael remained hospitalised for half a year, being released only after showing repentance and a willingness to regenerate. On the other hand, Manuel, diagnosed with paraphrenia and whose time in the sanatorium was brief, did not need to show any regret for shooting the priest, nor did he need to show a willingness to regenerate and avoid this kind of behaviour to be released.

The trajectories of these individuals presented side by side in this text differ significantly and are brought together here to problematise the possibilities and limits faced by specific individuals in the face of knowledge and powers, such as those of medicine or families. The institutional histories of Manuel and Natanael are used as examples to reflect on the emotions related to the experiences, both past and present, recorded in their manuscripts. I seek to understand the meanings these emotions possibly had for these individuals and how they were employed as part of a game that involved emotional resistance and negotiation with those who, in the context of the prevailing emotional device, occupied a position of superior status concerning their voices.

## Manuel

While hospitalised at Pinel, Manuel wrote a letter addressed to ‘Mr. Director of the Pinel Sanatorium’. In thirty-one pages, in what was the longest of the letters found in the medical records, its author described the events that resulted in the assassination attempt on the priest from the town of Angatuba, in the interior of the state of São Paulo. His story was initially told orally to doctors Pacheco e Silva and Nelson de Toledo Ferraz, who examined him the day after he was hospitalised. The doctors allegedly won his ‘trust’ by promising they would work ‘to get him out of the hospital’. Pacheco e Silva wrote the examination, but Ferraz encouraged Manuel to put down on paper the story he had told them both ‘passionately and in great details’.^65^

Twenty days into his hospitalisation, on 7 May 1935, Manuel finished his letter, as indicated by the date at the end, accompanied by his signature. The text begins with an introduction of the author: ‘Manuel Feitosa Cardoso, Brazilian, pharmacist, married, 48 years old, living twice, in Angatuba where he has a pharmacy, and in Juiz de Fora, at Rua Manoel Bernardino 123, where he is bringing up his children.’ Manuel then describes aspects of his personal and professional life, including his marriage to Mercedes Veloso de Freitas, the daughter of an influential local politician. Initially prosperous, the marriage was marred by family conflicts and financial losses caused by his brothers-in-law. Manuel also reports an adultery case involving his wife and Father Afonso Moreira from the parish of Angatuba, a fact that was documented by his daughter, Magdalena. The letter highlights, in its first pages, the tensions between Manuel and his family members, as well as the accusations made against the priest, to whom he attributes the destruction of his honour, harming his life.^66^

Manuel, who says he feels unjustly deprived of his inheritance by his brothers-in-law, suspicious of his wife’s betrayal, and convinced without ‘any more doubts [about] who had made his two daughters miserable forever by prostituting their mother’, describes a sequence of intense emotions. He also believes that he is being pursued ‘by the priest’s men who are trying to murder him’ and conjectures that ‘she [his wife] knew about the sinister plot’. In addition, he recounts the sensation of being ‘hunted down like a beast by the hunters’, which he heard while walking along the ‘lining of his house’.^67^ With such circumstances, Manuel channelled his emotions through writing, undertaking a profound catharsis. Perhaps, ‘the cathartic sensation of letting out so much stored up, of exorcising ghosts from the past, killing them again dead on paper’,^68^ in response to the impossibility of directly confronting those who, real or imagined, caused him fear and suffering, allowed him to rework his own life at that moment.

Finally, Manuel stated that he felt confident in the institution that housed him, the Pinel Sanatorium, ‘which has a heritage to protect and whose social mission is very high’, to ‘bring the fact to the attention of the Investigations Delegate’, who was to request various measures, such as hearing key witnesses, investigating his home and obtaining statements from his wife and daughter about the priest’s involvement in acts of dishonour and threats to his life. Finally, Manuel calls on the Sanatorium to take responsibility and defend his honour and safety.^69^

Although the Pinel psychiatrists did not consider Manuel’s account plausible – described as ‘a well-systematised persecutory delirium’, in which he went so far as to include ‘doctors and nurses among his persecutors, as possible allies of the priest he had shot’ – or perhaps precisely for this reason, they helped to ensure that the ‘deprivation of senses’ argument was used in his defence. Two letters from Juiz de Fora after Manuel left the sanatorium indicate that the doctors issued a medical report and attached it to the case brought against him.

In the first letter, dated 4 June 1939 and addressed to ‘Professor Pacheco e Silva’, Manuel mentions the imminence of the trial in Itapetininga. He states his plan to seek the report at the Sanatorium. He says he intends to be ‘more discreet’ in his ‘statements’ and to use the argument of ‘deprivation of senses’ in his defence. The letter ends with reaffirming his confidence in Pinel’s support: ‘My conception of reason goes today as far as sincerity exists, beyond that is imprudence, falsehood always prevails. So, I can count on Pinel’s patronage.’^70^

The second letter, dated 27 October 1941 and sent to ‘Dr Nelson de Toledo Ferraz’, shows that Manuel was re-established professionally, as it uses the letterhead of ‘*Farmácia Santa Honória, do Farmacêutico Manuel Feitosa Cardoso*’, in Juiz de Fora. In this letter, he expresses his gratitude to the Pinel doctors for his freedom and the recovery of his civil rights, mentions the difficulties he faced in the court case in Itapetininga, and celebrates the victory he won. He also affirms his commitment to the fight against endemic diseases and makes himself available to the doctors to repay their help.^71^

From a medical point of view, this letter, written more than two years after his hospitalisation, shows various signs of persecutory delirium, as evidenced by passages such as: ‘Our struggle was fierce, and we won proudly, even though the burglars of my house, my aggressors and those who stole my degree ring, money and jewellery from the house were informed.’^72^

What would have allowed Manuel, diagnosed with paraphrenia, to re-establish himself professionally and, as this letter indicates, escape both the ban and the sentence for the attack on the priest?

The articulation of specific social markers may explain this. Manuel, being a man with a certain level of material possessions, a recognised profession, a family man, and a respectable citizen, seems to have had his aggression – motivated mainly by the suspicion of his wife’s adultery with the parish priest – overlooked by the instances of knowledge and power that surrounded him, such as psychiatry and justice. Although his act was reprehensible and he was, at one point, characterised as a ‘dangerous stalker’, it was also considered justifiable because he had been driven by intense emotion due to the suspicion of adultery, which had deprived him of his reason and made him act outside normal standards. It is important to note that ‘deprivation of the senses’ was recognised as a mitigating factor in crimes against life, as provided for in Brazil’s Republican Penal Code of 1890, which was still in force. Article 27, item IV, stated: ‘Those who are in a state of complete deprivation of senses and intelligence at the time of committing the crime are not criminals.’^73^ As numerous cases discussed in different studies show, this argument was widely used in the defence of defendants accused of crimes considered to be ‘crimes of passion’, who were not seen as being as dangerous as other common offenders with ‘perverse instincts’. Even though Manuel did not direct his aggression directly at his wife – whom he also threatened to kill – but at her alleged lover, it is possible that his behaviour was framed as a ‘crime motivated by passion’.^74^

Or, at least, that was the narrative that Manuel skilfully constructed to support his defence. It is impossible to deny that he showed skill in handling speeches – he knew how to play the game – by arguing that his actions were motivated by intense emotions that deprived him of his senses. By choosing to ‘be more discreet’ in his ‘statements’ – adjusting, albeit momentarily and tactically, his most intense emotions – Manuel was able to rebuild his life and regain his social position.

## Natanael

As mentioned earlier, the young Natanael Borges was admitted to the Pinel Sanatorium at the request of his father, who was responsible for applying for admission, accompanied by a medical certificate signed by ‘two medical officers, Dr Torres Neto and Dr Pedro Augusto da Silva’.^75^ Designed by Pacheco e Silva, Natanael’s Psychiatric Examination included information provided by his family, which substantiated the justification for his admission. Based on this information, the doctor described Natanael as a dedicated and hard-working young man who founded the *Liceu Acadêmico Belo Horizonte* (Belo Horizonte Academic Lyceum) with the help of his sister. She was later removed from the administration, and the management was entrusted to ‘Professor José Carlos Flores, who had absolute control over him’ and with whom he began to live and share the same room. The family, unhappy with this situation, interpreted their living together as an indication of homosexual practices. Faced with unsuccessful attempts to change their son’s behaviour – considered ‘depressing and humiliating not only for Mr Natanael but for the whole family’ – they opted to have him admitted to the sanatorium. According to the doctor, the parents attributed such behaviour to a ‘morbid perversion’, in clear contrast to the strict upbringing and traditional moral values they had passed on to their son.^76^

Although the report alludes to the initial moment of hospitalisation, it seems to have been written at the end of the hospitalisation period, considering the doctor’s mention of the ‘long waiting time for the interdiction mental examination’, during which Natanael remained hospitalised, as well as the observations about his behaviour throughout this period.

According to Pacheco e Silva, ‘in this Sanatorium, Mr Natanael has not shown any sensory disturbances; however, he has shown himself to be mannered, affected and effeminate.’ In addition, he ‘denies the facts of which he is accused, but does so without vehemence, insisting that his business be entrusted to his partner and not to his family, as can be deduced from the attached letters’. The examination also identified ‘some deficiencies in critical judgement and, above all, a certain moral insensitivity’, evidenced by Natanael ‘talking naturally about the reasons that brought him to this Hospital’. The doctor writes that, during the ‘long waiting time for the mental examination to be interdicted’, Natanael initially showed revolt at his hospitalisation. However, when he realised that his and his partner’s efforts to get him released had been ‘unsuccessful, he settled for the situation’. Apart from ‘a certain apathy and indolence’, the doctor did not identify any other relevant aspects during his stay. Finally, he states that Natanael declared that he was ‘sorry for his behaviour and willing to regenerate’, seeking a reintegration into ‘ordinary life’.^77^

The medical opinion clearly shows the conception that characteristics such as being ‘mannered, affected, effeminate’ were signs of a psychological abnormality, reinforced by the lack of ‘critical judgement’ and ‘moral insensitivity’ observed in the way Natanael talked ‘naturally’ about such issues. The bodily and behavioural signs – such as the fact that he lived with his ‘friend’ and wanted him to run ‘his business’ – were interpreted by Pacheco e Silva as confirmations of the family’s allegations, which attributed to the young man a ‘morbid perversion’.

On the other hand, Natanael’s clear expression of his understanding of the reasons that led to his hospitalisation can be understood as a result of his trust in the doctor assessing him. Natanael may have perceived in the psychiatrist, who was also relatively young and an enlightened scientist, a potential ally, capable of understanding his identity without making moral judgments. In other words, someone who could stand against his father’s ignorance or intransigence. So, Natanael rebelled, argued, spoke openly, and then wrote about what he was experiencing. However, this was a mistake on his part.

As the bibliography shows, ever since he began his professional practice at the Juquery Hospital – a pattern he maintained in the other institutions where he worked as a doctor or professor – Pacheco e Silva always pathologised sexual practices that deviated from the heteronormative standard and that broke the limits of marriage. These practices were ‘considered “abnormal” by him and subject to intervention by psychiatrists’.^78^

Like the doctor, Natanael’s mother showed no solidarity with his demands, as evidenced by the letters he wrote to ‘Dear *Professor José Carlos Flores*’, whom the family identified as his lover.

In the first letter, written shortly after he was admitted to the sanatorium, Natanael is convinced that his mother was an ally in his resistance to hospitalisation and would act as an intermediary to keep José Carlos Flores in charge of the Lyceum. Prevented by ‘internal regulations’ from communicating directly, he informs him that the letter – which never reached its addressee – was being sent via a person called ‘Mr Ferdinando’. The aim was for Flores, armed with the correspondence, to present himself ‘as soon as possible at the Lyceum, so that he could take care of it’ with his mother.^79^

In the message, Natanael recalls the existence of a document he signed, emphasising its judicial value, which would guarantee José Carlos the right to take part in the running of the Lyceum. It also gave him the authority to supervise the financial administration of the institution, including the collection of tuition fees and the payment of uniforms for students on the Brazilian Army’s reservist training course. He then informs him that his mother had already made the payments and that he had come to an understanding with her, planning to later clarify to his father ‘the way you should be treated at the lyceum’. Natanael tells José Carlos that he can continue to pay the rent for the ‘room in Rua Oriente’, stay there if he prefers or move to another location. He also assures him that the teacher should continue to receive his salary. However, the document does not specify the exact amount, which suggests that previous payments could have been variable.^80^ In the final section of the letter, Natanael encourages Flores, whom he refers to as ‘your friend’, to remain in charge of the Lyceum:

Flores, come without fear because you have the full right and duty to attend once you’ve continued with my declaration. I have made a deal with Mum, and to avoid anything, she will only go to the Lyceum. My brothers will be independent of the movement, i.e. the Lyceum. I’m asking you to listen to Mum because she’s really kind. I’m the one who made a big mistake. As soon as I arrive at the Lyceum, maybe you’ll take over as headmaster, and I’ll go and study medicine.^81^

Natanael says goodbye, expressing how much he misses everyone, especially José Carlos, and wishes to be visited by him in the sanatorium’. Not knowing that his correspondence would never leave the sanatorium, he said that he would remain hospitalised out of respect for his parents’ wishes ‘until they see fit’. Natanael also instructs José Carlos Flores to continue ‘working quietly for the progress of the lyceum’, as if he ‘were there’.^82^

On 25 January, Natanael wrote another letter to José Carlos again, believing it would reach the addressee. This time, he emphasised the need for discretion: he asked him to avoid telephoning or sending correspondence and to ensure no one knew about their communication. He justifies this by saying that if ‘they find out that people outside the family know I’m here, they might send me somewhere else’. He adds: ‘Don’t even tell anyone how the letters came about.’^83^

This second letter has a less tranquil tone than the previous one. In the first letter, Natanael shows that he believes in his mother’s goodness and complicity, maintaining an attitude of respect and consideration for his parents despite everything. In the second, he shows fear and revolt. Addressing José Carlos as ‘Friend and brother Flores’, Natanael insists he ‘does not lose heart’, blaming the family for ‘everything that’s happening’. He encourages the ‘friend’ to react, ‘whatever it takes’, in the face of the difficulties related to the family’s rejection of his alleged lover’s presence at the Lyceum, even with the ‘necessary authorisation’ granted by him. In addition, he advises Flores to send the authorisation to a lawyer and mentions that he had sent information about her ‘case’ and ‘situation’ via someone called ‘Zezinho’. A string of emotionally charged words appears at the end of the short letter: ‘Flores, I can’t bear the longing, but I dare to suffer and fight on, whatever the cost. Only death will separate us. Yours, your brother and friend.’^84^

Natanael’s third and final letter was written on 20 March 1935 and addressed to his ‘kind mother’. Before succumbing to ‘apathy and indolence’, as described by Pacheco e Silva, and abandoning his resistance to the hospitalisation and removal of José Carlos Flores, whom he wanted to see in charge of his business, Natanael confronted his mother. This maternal figure, who he initially believed to be his ally against what he considered to be absurd – his hospitalisation and interdiction – became the focus of his protest. The narrative highlights Natanael’s internal conflict in the face of the rupture of his expectations regarding family support while simultaneously exposing his struggle to remain connected to what he considered essential in his life.

The letter indicates that Natanael was somehow able to act in his defence, either through his lawyer or through the intervention of José Carlos, with whom he established contact through other means, since his letters had been retained. In his narrative, he contests the accusations that resulted in his deprivation of liberty, his removal from his professional activities, and from the man he called ‘brother’ or ‘friend’. The accusations refuted by Natanael appear to have been presented in a letter sent to him by his mother, but this correspondence is not archived in his medical records.

The letter, written in impeccable handwriting, begins affectionately, with Natanael expressing ‘how much I miss you’ and congratulating his mother on her birthday. Quickly, however, the tone of the correspondence changes, showing a more defiant attitude, as he expresses, in a frank and resolute manner, the difficulty of meeting the recipient’s expectations: ‘I’m sorry I can’t be pleasant to you in reply to the letter that comes with your name.’ The following statement reveals her willingness to confront the feelings involved directly: ‘My intellectual and moral culture, thanks to your efforts, cannot come up with a fictitious sentimentality to make you simply happy.’^85^

Nathanael then justifies his decision not to reply directly to the letter he received, as this would mean disagreeing with specific facts described in it, setting out his ‘reasons’ and therefore disagreeing with ‘certain facts described in it such as: Friendly illusion. Uncertain routine. Medical daring. Police trickery. Role of Iscariot’. Although he refuses to detail the points of disagreement, Natanael anticipates his intention to spare the addressee unnecessary inconvenience, avoiding ‘upsetting and disturbing her’. At the same time, he seeks to justify his behaviour and attributes a greater purpose to it when he says, ‘I, armed with an iron will, will be the safe rudder on the great ocean of life, thus achieving the name that will make my family proud.’ The use of these metaphors emphasises the author’s self-confidence at this time in overcoming the adversities imposed on him.^86^

A legal issue is addressed next in the letter, with Natanael taking a stance to dispel another accusation made by his mother, that of a supposed lawsuit against his father: ‘That letter talks about a lawsuit against Dad, but it is not a lawsuit against him, but a request for Habeas Corpus that I made to the judge.’ The request for Habeas Corpus was based on the circumstances in which the police had arrested him and subsequently interned at the Pinel Institute, which he considered to be unjust and carried out in degrading conditions: ‘I was arrested by the police and brought to the Pinel Institute, where I was interned in unpleasant conditions that could not be attributed to me: mental disorders and homosexual tendencies.’ Natanael vehemently rejects these attributions, arguing that they were based on other people’s statements – ‘the first by Papa, who attached a medical certificate, and the second by Dr Pacheco, Director of the Pinel Institute’ – and not on concrete facts. Not admitting these accusations and protesting against an internment that had even left him ‘incommunicado’, Natanael states that ‘in his right mind, understanding and health’, considering his ‘internment in the Sanatorium to be “illegal” (as approved by the Public Prosecutor Dr Ataliba Nogueira)’, he had appealed ‘for justice’.^87^

In the last paragraph of the letter, the author states that he believed ‘that he could leave the Sanatorium with the same politeness’ with which he had entered it, because the ‘false label of rest’ that had justified his hospitalisation, expelling him ‘out of the blue, from the (…) Lyceum of which he was Director and from the Presidency of T. J. 20’, had been based on ‘false and very inconstant claims’. In his words, these had caused his father to avoid ‘sensibly analysing the truth’ and to be ‘forced to proceed in the way that happened’. At this point, there is no confrontation but rather an attempt at negotiation by convincing people of the existence of reciprocal errors, which would have resulted in the hospitalisation. It was this, according to Natanael’s argument, and not a challenge to the family order or social and gender norms, that engendered the events. That was why he appealed to the courts and waited for their decision, as he writes in his letter.^88^

Natanael closed his letter by saying to his mother, ‘What I want is for you to remain calm, as well as everyone at home.’ He thus reiterates his affection and concern for the well-being of his family, to whom he sends his ‘regards’.^89^

Denying the main accusation that had motivated his hospitalisation at Pinel and, consequently, his removal from his business employing a civil interdiction, which I’m not sure was carried out, Natanael tried to play the game that had been imposed on him. Not believing in any partnership, at the level of understanding and acceptance, on the part of his doctor, he dismissed the statements he said he had made about his homosexuality, calling the statements of both the doctor and his father confusing and divergent. Far from a ‘fictitious sentimentality’, but seeking to maintain his ‘politeness’, a gentle attitude that expresses a positive emotion, contrary to the perverted or depraved ones attributed to those who engaged in homosexual practices, Natanael appealed for his rights in the letter mentioned, as I have indicated.

However, none of this seems to have worked. His legal and personal appeals had no effect because, as Pacheco e Silva reported, ‘his efforts and those of his partner who was interested in his leaving had been fruitless’. Natanael only left the sanatorium when he ‘came to terms with the situation’, promising to adjust to family and medical expectations about his sexuality, saying he ‘regretted his behaviour’. What happened seems to have been an act of resistance in accommodation, in the same way Dolores had acted. It was an adjustment to the prevailing emotional device, which involved denying his sexual orientation. By claiming to be ‘willing to regenerate’, at least in front of those who had the authority to ‘give him his ordinary life back’ – his doctor and his family – Natanael won his freedom.^90^

But what kind of freedom was this? Certainly not the one desired by Natanael, in which love for an individual of the same sex – clearly expressed in the second letter addressed to João Carlos – was not considered an abnormal emotion.

Homosexuality would increasingly become a psychiatric problem, with all emotions and practices related to it being considered abnormal. In 1948, it became part of the International Statistical Classification of Diseases and Related Health Problems (ICD). In the sixth revision, ‘homosexuality’ was included in subcategory 320.6 – Sexual Deviation. From then until 1990, when the World Health Organisation removed it from the ICD, ‘homosexuality’ was maintained in the successive revisions that this type of manual underwent.^91^

The inclusion of the ‘homosexuality’ in the International Classification of Diseases (ICD) and the Diagnostic and Statistical Manual of Mental Disorders (DSM) of the American Psychiatric Association (APA) reinforced the convictions of Pacheco e Silva who, from the time of the events studied, remained active in combating practices and emotions that he considered to be deviations from human nature. For the psychiatrist, social stability was linked to the preservation of sexual morality, respect for ‘chastity, virginity, pudency, decency, marital fidelity, and normal sexual relations’. In this sense, he argued, civilised societies had always combated promiscuity and ‘sexual perversions, such as homosexuality’, a true ‘aberration of nature’.^92^

Had Natanael somehow managed to circumvent the rigid restrictions imposed on him, restrictions marked by episodes of profound violence, as evidenced by his hospitalisation and attempted judicial interdiction? Had he and João Carlos resumed their love affair, albeit under the cloak of clandestinity?

Although this hypothesis is plausible, its counterpart cannot be overlooked: the possibility that family and social pressures continued to exert force on the once daring Natanael. In a past of freedom, he had been enterprising and had dared to share his life with a partner of the same sex. Have the intense emotions that once pulsed through Natanael, such as passion and obstinacy, been replaced by others of an opposite nature, such as fear and resignation?

The emotional resistance visible in his letters, exposed in a harsh or negotiated way, may have given way in the face of obstacles that Natanael was unable to overcome. Thus, entangled in the intricate webs that imprisoned him, he may have become the apathetic and resigned person in Pacheco e Silva’s account, an emotional stance that the psychiatrist seems to be proud of, as it indicates that the disciplining power of institutional practices affected him. However, if this were the case, would such a transformation be definitive, marking the course of Natanael’s existence forever?

He did not return to the Pinel Sanatorium, which became a public institution a few years after his visit. Still, perhaps he went on to other spaces for disciplining bodies and minds, aimed at the wealthier classes in the city of São Paulo.

## Conclusion

Marília, Valentina, Dolores, and Luísa – mentioned in this text – as well as Helena, Maria Antônia, and Heloise, referred to as ‘domestics’ or without any record of their profession, are the women whose letters I found in the Pinel archives. Aldemir, Valentim, Paiva, Dr Jorge, and Dr Frederico are some of the other twenty men who, along with the aforementioned Lúcio, Manuel, and Natanael, made up the population of those who left records of their time at the Sanatorium. Between the late 1920s and the 1940s, these men with different occupations – simple farmers, teachers, lawyers, and engineers, whose surnames were streets in the expanding metropolis – and the women were part of a ‘hierarchical emotional device’. This device was structured by ‘strategies, technologies, coercions and persuasions’, which involved various social groups, such as families and psychiatrists, as well as institutions that believed in the function of the psychiatric institution as a tool to ‘reconduct and reform’ behaviours that were considered disruptive and emotions that were out of the norm.^93^

They also actively participated in this mechanism, sometimes adapting and sometimes resisting. The ‘emotional resistance’ outlined in the relationships in the hospital sometimes took the form of negotiation or resistance to accommodation, as when Dolores told the psychiatrist she had ‘good intentions for the future’ or when Natanael said he was ‘willing to regenerate’. But on many other occasions, ‘emotional resistance’ is expressed more vigorously, through escapes announced in correspondence, in writings that despise or mock medical assessments; in others that report complaints of mistreatment outside and inside the institution, promising to denounce the injustices experienced in the newspapers; or even through suicide, an extreme line of escape, as Lúcio did. Emotional resistance, as Rosón and Medina Doménech say, ‘does not have to be completely innovative, but it does show us a creative human conscience that is worth rescuing (…), since it can be read as a “repository” of knowledge, a “repository” of good living.’^94^

Both the medical reports in the Psychiatric Examination and the content of the letters reveal, albeit from different perspectives – sometimes highlighting the abnormality, sometimes emphasising the potential of the subjects –, the movement of emotions and the play with them. Emotions, far from being simple substances in ‘our blood’, are social practices organised by stories we act out and tell. Our ways of understanding structure them,^95^ as I have endeavoured to demonstrate throughout this text.

On the other hand, as I pointed out right from the start, the social markers that constructed the subjects, visible both in the trajectories I have briefly outlined here and in those of the other people who wrote letters, continued to fulfil their role in establishing equalities and differences within the modern sanatorium. All the interned people were equalised along three main lines: the condition of being suspected of being mad – with or without a defined diagnosis –; under their more privileged social status – which allowed them to pay for their stay in a private institution –; and by their ‘ethnic data’ declared on the internment form – white. However, they were differentiated when the family, then medical knowledge, evoked the prevailing standards and norms of gender and sexuality in the definition of normality/abnormality.

In the Pinel Sanatorium, as in other similar institutions – widely analysed by the historiography of madness and psychiatry, as well as by gender studies, especially those focused on mental institutions – the social markers of difference had a more significant impact on women than on men. Women’s hospitalisation was often justified by the so-called emotional excesses inherent in female nature, as in the case of Helena, described as ‘spectacular, with theatrical attitudes’, ‘rebellious’ and ‘extravagant’.^96^ In addition, the breaking of moral norms associated with the wife/mother/housewife triad was also the reason for these hospitalisations, as exemplified by the case of Marília, who was involved in a ‘violent scene of blood (…) killing her lover with axes and gunshots.^97^

In this sense, complaints about physical, psychological, moral, sexual, or property violence by their male partners were disregarded. On the other hand, a man’s violence, motivated by so-called ‘passion’, could act as a mitigating factor for his madness, if it was understood as a temporary ‘deprivation of the senses’ or the result of a ‘motor impulse’ followed ‘immediately’ by repentance, as in the cases of Manuel and Lúcio. However, if a man was openly homosexual – like Natanael – there was little chance that he would not be considered to have a ‘pathological personality’, especially if such an orientation became public, jeopardising the reputation of well-established families in local society.

## Footnotes

1 All names are pseudonyms of people admitted to the Pinel Sanatorium, whose medical records are analysed in this text and duly referred to below.

2 The term ‘civil ban’ refers to the idea of ‘civil interdiction’. The term ‘civil interdiction’ designates a juridical measure codified in Law no. 3,071 of 1 January 1916, the Brazilian Civil Code operative at the time. This provision, found in the Book of Family, permitted the legal incapacitation of individuals evaluated as absolutely or relatively incapable of the civil acts pertaining to self-governance and administration of property. Classes subject to interdiction included the deaf-mute, the prodigal, and, explicitly, all persons classified as ‘madmen of every kind’ or ‘insane of all kinds’, reflecting the technical nomenclature prevalent in early twentieth-century Brazilian law. Brazil, Civil Code, Law no. 3,071, 1 January 1916, arts 446–447.

3

Heloisa Starling and Lilia M. Schwarcz , ‘Lendo canções e arriscando um refrão’, Revista USP, 68 (2005-2006), 210–233 .

4

Heloísa Almeida et al, ‘Numas, 10 Anos: Um Exercício de Memória Coletiva’, in Gustavo Santa Roza Saggese et al. (ed.), Marcadores Sociais Da Diferença: Gênero, Sexualidade, Raça e Classe Em Perspectiva Antropológica (São Paulo: Terceiro Nome; Gama, 2018), 19 .

5

Barbara Rosenwein , ‘How to Practice the History of Emotions’, Brathair, 23, n.1 (2023), 39, 40.

6 Lúcio. Doc. no. 1295, tin CO9610, Year 1935. Arquivo Público do Estado de São Paulo (hereafter APESP), Fundo Sanatório Pinel.

7 Luísa. Doc. no. 1020, tin CO9603, Year 1934. APESP, Fundo Sanatório Pinel.

8 Antonio Ackel Barbosa, ‘Cartas Pessoais de Pacientes Do Sanatório Pinel (1929–1944): Um Estudo Filológico’ (unpublished Master’s thesis: University of São Paulo, 2019).

9 Barbosa’s research found 42 texts (letters addressed to someone, memoirs written at the doctor’s request, short texts that look like poetry or just notes, one of them containing a drawing), written by 30 people hospitalised at the Pinel Sanatorium, in the period indicated. I took as a reference the survey carried out by the author for my own research on the psychiatric records whose data will be problematised here. The documentation is held by the Public Archives of the State of São Paulo (APESP) and is part of the Pinel Sanatorium Fund. Cf. Barbosa, *op. cit.* (note 7).

10 Yonissa Marmitt Wadi, *A História de Pierina: Subjetividade, Crime e Loucura* (EDUFU, 2009); Olga Villasante et al., ‘Letras retenidas. Experiencias de internamiento en las cartas de los pacientes del Manicomio de Santa Isabel de Leganés, Madrid (1900–1950)’, *Revista Culturas Psi/Psy Cultures*, n.6 (2016), 118–37, online: <http://ppct.caicyt.gov.ar/index.php/culturaspsi/article/view/8328/pdf_1>.

11 Gustavo Querodia Tarelow, ‘Antonio Carlos Pacheco e Silva: Psiquiatria e Política em uma Trajetória Singular (1898–1988)’ (unpublished PhD thesis: University of São Paulo, 2018).

12 Rita Cristina de Medeiros Couto, ‘Eugenia, Loucura e Condição Feminina’, 90, *Cadernos de Pesquisa*, 90 (1994), 52, online: <https://publicacoes.fcc.org.br/cp/article/view/892>, accessed 23 December 2023. See also: Rita Cristina de Medeiros Couto, ‘Eugenia, Loucura e Condição Feminina no Brasil. As Pacientes do Sanatório Pinel de Pirituba e o Discurso dos Médicos e dos Leigos Durante a Década de 1930’ (unpublished PhD thesis: University of São Paulo, 1994).

13 Tarelow, Antonio Carlos Pacheco e Silva, *op. cit.* (note 10), 233.

14 Ewerton Luiz Figueiredo Moura da Silva, ‘Do Sonho à Loucura: Portugueses e a Doença Mental Em São Paulo (1929–1939)’ (unpublished Master’s dissertation: Federal University of São Paulo, 2015), 86–7.

15 I would highlight the work of historian Gustavo Querodia Tarelow: Tarelow, Antonio Carlos Pacheco e Silva, *op. cit.* (note 10); Gustavo Querodia Tarelow , ‘A Moral, a Política e Algumas Questões Científicas Presentes No Hospital Do Juquery: Abordagens de Pacheco e Silva (1923–1950)’, Revista Histórica, 51, 7 (2011), 1–12 .

16 Maria Izilda Santos de Matos and Bruna Beserra Pereira, ‘“Antes Louca do que Menos Digna”. Prontuários femininos do Sanatório Pinel (São Paulo/Br. 1929–1944)’, *Arenal. Revista de Historia de las Mujeres*, 28, 2 (2021), 523, online: <https://revistaseug.ugr.es/index.php/arenal/article/view/15734>, accessed 23 December 2023. See also: Maria Izilda Santos de Matos and Bruna S. Beserra Pereira, ‘Prontuários Femininos do Sanatório Pinel/SP (1929–1944)’, *Revista Estudos Feministas*, 30 (2022), e76044, online: <https://www.scielo.br/j/ref/a/jfhwL3563CrLtbyYJsyFpFQ/?lang=pt>, accessed 23 December 2023. There are also several master’s dissertations that explore the same sources on women with issues very similar to those addressed in the works cited.

17 Tuanny Folieni Antunes Lanzellotti, ‘Controle e Internação Entre o Público e o Privado: Hospital Psiquiátrico Do Juquery e Sanatório Pinel De Pirituba (1929–1934)’ (unpublished Master’s dissertation: Federal University of São Paulo, 2018), 71.

18 Some examples are Silva, *op. cit.* (note 13); Nelson Tomelin Jr., ‘A Cultura Da Loucura Na Cidade: O Planejamento Da Velhice Saudável e a Esperança Que Falou (São Paulo, 1940/2005)’ (unpublished PhD thesis: University of São Paulo, 2008).; Nelson Tomelin Jr, ‘Planejamento Manicomial. A Produção Social da Loucura (Hospital Psiquiátrico Pinel, São Paulo/1940)’, *Projeto História: Revista do Programa de Estudos Pós-Graduados de História*, 38 (2009), 205–19, online: <https://revistas.pucsp.br/index.php/revph/article/view/5240>, accessed 23 December 2023.

19 The following works are examples of this discussion: Rita Cristina Carvalho de Medeiros Couto, *Nos Corredores Do Pinel: Eugenia e Psiquiatria* (São Paulo: LiberArs, 2020); Gustavo Querodia Tarelow and André Mota , ‘Antonio Carlos Pacheco e Silva: Eugenia, Raça e a Liga Paulista de Higiene Mental, 1926–1950’, Projeto História: Revista do Programa de Estudos Pós-Graduados de História, 75 (2022), 148–68, online: <https://revistas.pucsp.br/index.php/revph/article/view/58645>, accessed 23 December 2023.

20

Maria Célia Lima-Hernandes , ‘Prontuários Médicos e o Corpus Do Português de São Paulo: Vestígios Da História Social Feminina Na Primeira Metade *Do* Século XX’, Anais Do XV ALFAL, Vol. 1 (Montevideo: ALFAL, 2008); Barbosa, *op. cit.* (note 7).

21 Roy Porter, ‘The Patient’s View: Doing Medical History from Below’, *Theory and Society*, 14, 2 (1985), 175–98, online: <https://www.jstor.org/stable/657089>, accessed 26 December 2023; Roy Porter, *Uma História Social da Loucura* (Rio de Janeiro: Jorge Zahar Editor, 1991).

22 Porter, The Patient’s View, *op. cit.* (note 20); Porter, *Uma História Social*, *op. cit.* (note 20); Wadi, *op. cit.* (note 9); Villasante et al., *op. cit.* (note 9); Rafael Huertas , Locuras en Primera Persona (Madrid, Cataratas, 2020); Rafael Huertas , Historia Cultural de la Psiquiatría. (Re)pensar la Locura (Madrid: Catarata, 2012); Yonissa Marmitt Wadi et al., ‘¿Qué Expresan los Locos Iberoamericanos? Las Fuentes Narrativas y sus Posibles Abordajes’, Iberoamericana, 19, 71 (2019), 173–95, online: <http://doi.org/10.18441/ibam.19.2019.71.173-195>, accessed 29 June 2021; Yonissa Marmitt Wadi, ‘“Entre Muros”: os Loucos Contam o Hospício’, *Topoi (Rio de Janeiro)*, 12 (2011), 250–69, online: <https://www.scielo.br/j/topoi/a/MxrYq3g7HvnzKMsvzbZVvRf/?lang=pt>, accessed 29 June 2021.

23 A review of Ibero-American historiography that uses narratives of mad people as sources can be found in Wadi et al., *op. cit.* (note 21).

24

Rafael Huertas , ‘Another History for Another Psychiatry. The Patient’s View’, Culture & History Digital Journal 2, n. 1 (2013), e020 , online: DOI: http://doi.org/10.3989/chdj.2013.020, accessed 29 June 2021.

25 Oliva López Sánchez, ‘La Pertinencia de una Historia de la Construcción Emocional del Cuerpo Femenino en México entre 1850–1910. Abordaje desde el Construccionismo Social’, *Revista Latinoamericana de Estudios sobre Cuerpos, Emociones y Sociedad* 5, 12 (2013), 58, online http://relaces.com.ar/index.php/relaces/article/view/359, accessed 19 November 2024.

26 María Rosón and Rosa Medina Domenech, ‘Resistencias Emocionales. Espacios y Presencias de lo Íntimo en el Archivo Historico’, *Arenal*, 24, 2 (2017), 407–439, online https://revistaseug.ugr.es/index.php/arenal/article/view/3914, accessed 19 November 2024.

27

Fay Bound Alberti , ‘Introduction: Medical History and Emotion Theory’, in Fay Bound Alberti (ed.), Medicine, Emotion and Disease, 1700–1950 (New York: Palgrave Macmillan, 2006), xiii–xxviii .

28

Rosa María Medina Doménech , ‘Sentir la Historia. Propuestas para una Agenda de Investigación Feminista en la Historia de las Emociones’, Arenal. Revista de Historia de las Mujeres 19, 1 (2012), 161–199 , online https://doi.org/10.30827/arenal.v19i1.1412, access 19 November 2024; López Sánchez, *op. cit.* (note 24).

29

Oliva López Sánchez and Margarita Gutiérrez Colín , ‘Los Roles de Género y la Expresión Emocional, Elementos Fundamentales en el Diagnóstico de la Locura: el Caso de Lucía N. de Ortiz Moradora de La Castañeda de 1937 a 1952’, in Oliva López Sánchez (ed.), La Pérdida del Paraíso. El Lugar de las Emociones en la Sociedad Mexicana entre los Siglos XIX y XX. (Ciudad de México: Universidad Nacional Autónoma de México, 2011).

30 Barbara Rosenwein, ‘II. Barbara H. Rosenwein’, in Jan Plamper , ‘The History of Emotions: An Interview with William Reddy, Barbara Rosenwein, and Peter Stearns’, History and Theory 49 (May 2010), 237–265 .

31 Some authors who question and justify the use of a restricted documentary corpus are Arlette Farge, *Le Goût de l’Archive* (Seuil, 1989); Carlo Ginzburg, *Myths, Emblems, Clues* (Hutchinson Radius, 1990); Wadi, *op. cit.* (note 9); Rafael Huertas, *Locuras en Primera Persona op. cit.* (note 21).

32

Teresa Ordorika and Aída Alejandra Golcman , ‘Diagnóstico: Histeria. Abordando Histórias Clínicas del Manicomio General la Castañeda’, in Teresa Ordorika and Aída Alejandra Golcman , Locura en el Archivo. Fuentes y Metodologías para el Estudio de las Disciplinas Psi (Mexico: Universidad Nacional Autónoma de México, Centro de Investigaciones Interdisciplinarias en Ciencias y Humanidades, 2021), 89 .

33 Rafael Huertas, *Historia Cultural*, *op. cit.* (note 21).

34 Porter, ‘The Patient’s View’, *op. cit.* (note 20); Roy Porter, *Uma História Social*, *op. cit.* (note 20).

35 Frida Gorbach, ‘¿Dónde Están las Mujeres de La Castañeda? Una Aproximación a los Expedientes Clínicos del Manicomio, 1910’, *Nuevo Mundo Mundos Nuevos. Nouveaux mondes mondes nouveaux – Novo Mundo Mundos Novos – New World New worlds* (2011), online: <https://journals.openedition.org/nuevomundo/61046>, accessed 14 February 2024; Dedier Norberto Marquiegui, ‘Los Archivos de la Locura en la Colonia Nacional de Alienados de Open Door. Preguntas Formuladas desde la Óptica de un Historiador’, 3, *Revista Electrónica de Fuentes y Archivos*, 3 (2012), 286–301, online: <https://revistas.unc.edu.ar/index.php/refa/article/view/34094>, accessed 14 February 2024; Ana Teresa A. Venancio, ‘La Historia de los Documentos Clínicos Psiquiátricos: Entre la Serie Documental y la Singularidad de los Casos’, in Teresa Ordorika and Aída Alejandra Golcman *Locura en el Archivo. Fuentes y Metodologías para el Estudio de las Disciplinas Psi* (Mexico: National Autonomous University of Mexico, Centre for Interdisciplinary Research in Sciences and Humanities, 2021), 21–53; Yonissa Marmitt Wadi, ‘Las Narrativas de los Locos y la Historia de la Locura y la Psiquiatría’, in Teresa Ordorika and Aída Alejandra Golcman*, Locura en el Archivo. Fuentes y Metodologías para el Estudio de las Disciplinas Psi* (Mexico: National Autonomous University of Mexico, Centre for Interdisciplinary Research in Sciences and Humanities, 2021), 55–85; Wadi et al., *op. cit.* (note 22); Ordorika and Golcman, *op. cit.* (note 31).

36 Gorbach, *op. cit.* (note 34).

37 Célia Garcia-Diaz, ‘Entre Mujeres-Locas y Hombres-Psiquiatras: Asimetrías Emocionales en la sala 20 del Manicomio Provincial de Málaga (1909–1950)’, *Asclepio*, 75, 1 (2023), 3, e06-e06, online: <https://asclepio.revistas.csic.es/index.php/asclepio/article/view/1163>, accessed 14 February 2024.

38 Rosón e Medina Doménech, *op. cit.* (note 25), 409.

39 Long stays were not common – at least in the reports analysed here – due to the high costs of hospitalisation, but they often ended up being due to the numerous entries and exits of someone, as the records in the psychiatric records and the reference bibliography cited above show.

40 Oliva López Sánchez and Margarita Gutiérrez Colín, ‘Los Roles de Género y la Expresión Emocional, Elementos Fundamentales en el Diagnóstico de la Locura: El Caso de Lucía N. de Ortiz Moradora de La Castañeda de 1937 a 1952’, in López Sánchez, *op. cit.* (note 28), 176.

41
*Ibid.*, 176. Michel Foucault , O Poder Psiquiátrico (São Paulo: Martins Fontes, 2006).

42 Lúcio. Doc. no. 1295, tin CO9610, Year 1935. APESP, Pinel Sanatorium Fund.

43

Vavy Pacheco Borges , Tenentismo e Revolução Brasileira. São Paulo: Brasiliense, 1992 (São Paulo: Brasiliense, 1992); Stanley Hilton , A Guerra Civil Brasileira: História Da Revolução Constitucionalista de 1932 (Rio de Janeiro: Nova Fronteira, 1982).

44 Lúcio. Doc. no. 1295, tin CO9610, Year 1935. APESP, Pinel Sanatorium Fund.

45 Lúcio. Doc. no. 1295, tin CO9610, Year 1935. APESP, Pinel Sanatorium Fund.

46 Lúcio. Doc. no. 1295, tin CO9610, Year 1935. APESP, Pinel Sanatorium Fund.

47 Dolores. Doc. no. 1370, tin CO9612, Year 1936. APESP, Pinel Sanatorium Fund.

48 Dolores. Doc. no. 1370, tin CO9612, Year 1936. APESP, Pinel Sanatorium Fund.

49 Dolores. Doc. no. 1370, tin CO9612, Year 1936. APESP, Pinel Sanatorium Fund.

50 Dolores. Doc. no. 1370, tin CO9612, Year 1936. APESP, Pinel Sanatorium Fund.

51

Henrique Roxo , Manual de Psiquiatria, 2^a^ (Rio de Janeiro: Guanabara, 1925), 637 *apud* Pedro Felipe Muñoz and Allister A. Teixeira Dias , ‘Degeneração, Subalternidade e Favela: Anália, “Uma Mulher de cor Preta” no Rio de Janeiro Pós-Abolicionista’, 27, Revista Maracanan, 27 (2021), 194–221, 206, online: <https://www.e-publicacoes.uerj.br/maracanan/article/view/57276>, accessed 22 February 2024. I am not sure if it was Pacheco e Silva, or one of his collaborators at Pinel, who gave Dolores the final diagnosis, but it is important to emphasise that he – and possibly the group of doctors who worked with him – were supporters of many of Henrique Roxo’s perspectives. According to Tarelow, ‘In Pacheco e Silva’s publications it is possible to note the presence of Henrique Roxo’s work as an important theoretical reference’. Tarelow, Antonio Carlos Pacheco e Silva, *op. cit.* (note 10), 143.

52
*Ibid.*

53 In my sample of 30 psychiatric records, ‘undefined diagnoses’ totalled 23%, i.e. they were recorded in seven records – of four men and three women; while four patients (13%), all men, had no diagnosis at all.

54

J. Hoenig , ‘Esquizofenia. Seção Clínica’, in German E. Berrios and Roy Porter (eds), Uma História Da Psiquiatria Clínica – II: A Origem e a História Dos Transtornos Psiquiátricos (São Paulo: Escuta, 2012), 533–49; Trevor Turner , ‘Esquizofrenia. Seção Social’, in German E. Berrios and Roy Porter (eds), Uma História Da Psiquiatria Clínica – II: A Origem e a História Dos Transtornos Psiquiátricos (São Paulo: Escuta, 2012), 550–64.

55 Doc. no. 1370, tin CO9612, Year 1936. APESP, Fundo Sanatório Pinel; Lúcio. Doc. no. 1295, tin CO9610, Year 1935. APESP, Pinel Sanatorium Fund.

56 Stéphane Audoin-Rouzeau, ‘Apocalipses da Guerra’, in Alain Corbin et al, *História das Emoções.* Do Final do Século XIX Até Hoje (Petrópolis: Vozes, 2020), 291–312; Marcelo Santos de Abreu, ‘Luto e Culto Cívico dos Mortos: As Tensões da Memória Pública da Revolução Constitucionalista de 1932 (São Paulo, 1932–1937)’, *Revista Brasileira de História*, 31 (2011), 105–23, online: <https://www.scielo.br/j/rbh/a/cfZfLbNYG9DQKYMrqqMzLht/?lang=pt>, accessed 19 February 2024.

57 Antonio Carlos Pacheco e Silva, *Medicina Psicossomática em Ginecologia* (São Paulo: Faculdade de Medicina de São Paulo, 1950), 22–23, *apud* Couto, Eugenia, Loucura e Condição Feminina, *op. cit.* (note 11), 56.

58

Celia García-Díaz e Isabel Jiménez-Lucena , ‘Clasificando Mujeres: Diagnósticos Psiquiátricos y Subjetividad Femenina en el Manicomio Provincial de Málaga, España, 1909–1950’, História, Ciências, Saúde-Manguinhos, 30 (2023), e2023003 37018778 10.1590/S0104-59702023000100003PMC10395599, online: <http://www.scielo.br/j/hcsm/a/hMf3vPMrRVYmKhgwLX4MLBK/?lang=es>, accessed 10 April 2023.

59

Céli Regina Jardim Pinto , Uma História Do Feminismo No Brasil (São Paulo: Editoria Fundação Perseu Abramo, 2003).

60

Cristiana Facchinetti and Carolina Carvalho , ‘Loucas ou Modernas? Mulheres em Revista (1920–1940)’, Cadernos Pagu (2019), e195707 , online: <https://www.scielo.br/j/cpa/a/4sMbWpZGzJtBsbhWxzf5YRB/?lang=pt>, accessed 19 February 2024.

61 Jean Anyon, ‘Interseções de Gênero e Classe: Acomodação e Resistência de Mulheres e Meninas às Ideologias de Papéis Sexuais’, 73, *Cadernos de Pesquisa*, 73 (1990), 13–25, online: <https://publicacoes.fcc.org.br/cp/article/view/1093>, accessed 21 February 2024.

62 Dolores. Doc. no. 1370, tin CO9612, Year 1936. APESP, Pinel Sanatorium Fund. Matos and Pereira, when studying Dolores’ case in an article, highlight an issue that was undoubtedly decisive in understanding what I call this woman’s ‘resistance to accommodation’: ‘It should be noted that under the codes in force at the time, women had limited rights and had to remain under the tutelage and control of their father and husband. Marriage was identified as a contract, on the one hand, the husband was responsible for earning the money, and on the other, women had to obey the “head of the family” and dedicate themselves to the duties of the home and motherhood. The marriage bond was considered indissoluble; separation, when necessary, restricted future unions and divorced women were socially disqualified’. Matos e Pereira, Antes louca, *op. cit.* (note 15), 534. Cf. also: Andrea Borelli , Uma Cidadã Relativa: As Mulheres, as Questões de Gênero e o Direito Brasileiro – 1830/1950 (São Paulo: DC&C empresarial, 2010).

63 Natanael. Doc. no. 1126, tin CO9606, Year 1935. APESP, Pinel Sanatorium Fund; Manuel. Doc. no. 2305, tin CO9638, Year 1939. APESP, Pinel Sanatorium Fund.

64 Manuel. Doc. no. 2305, tin CO9638, Year 1939. APESP, Fundo Sanatório Pinel; Natanael. Doc. no. 1126, tin CO9606, Year 1935. APESP, Pinel Sanatorium Fund.

65 Manuel. Doc. no. 2305, tin CO9638, Year 1939. APESP, Pinel Sanatorium Fund. According to Barbosa, who transcribed Manuel’s letter, ‘Given the length of the text and the small number of corrections, it is possible to conjecture that it is an autograph copy’, i.e. the author must have copied the original precisely in order to make the corrections and adjustments he believed necessary. Barbosa, *op. cit.* (note 7).

66 Manuel. Doc. no. 2305, tin CO9638, Year 1939. APESP, Pinel Sanatorium Fund.

67 Manuel. Doc. no. 2305, tin CO9638, Year 1939. APESP, Pinel Sanatorium Fund.

68

Davi Arrigucci Júnior , Enigma e Comentário: Ensaios Sobre Literatura e Experiencia. (São Paulo: Companhia das Letras, 1987), 68 .

69 Manuel. Doc. no. 2305, tin CO9638, Year 1939. APESP, Pinel Sanatorium Fund.

70 Manuel. Doc. no. 2305, tin CO9638, Year 1939. APESP, Pinel Sanatorium Fund.

71 Manuel. Doc. no. 2305, tin CO9638, Year 1939. APESP, Pinel Sanatorium Fund.

72 Manuel. Doc. no. 2305, tin CO9638, Year 1939. APESP, Pinel Sanatorium Fund.

73

José Henrique Pierangeli , Códigos Penais Do Brasil: Evolução Histórica. (Bauru: Jalovi, 1980), 271 .

74

Evaristo de Moraes , Criminalidade Passional: O Homicidio e o Homicidio-Suicidio Por Amor (Em Face de Psychologia Criminal e Da Penalistica) (São Paulo: Saraiva & Cia, 1933); Luísa Nagib Eluf , A Paixão No Banco Dos Réus (São Paulo: Saraiva, 2002); Mariza Corrêa , Morte Em Família. Legal Representations of Sexual Roles (Rio de Janeiro: Graal, 1983).

75 Natanael. Doc. no. 1126, tin CO9606, Year 1935. APESP, Pinel Sanatorium Fund.

76 Natanael. Doc. no. 1126, tin CO9606, Year 1935. APESP, Pinel Sanatorium Fund.

77 Natanael. Doc. no. 1126, tin CO9606, Year 1935. APESP, Pinel Sanatorium Fund.

78 Tarelow, Antonio Carlos Pacheco e Silva, *op. cit.* (note 10), 271.

79 Natanael. Doc. no. 1126, tin CO9606, Year 1935. APESP, Pinel Sanatorium Fund.

80 Natanael. Doc. no. 1126, tin CO9606, Year 1935. APESP, Pinel Sanatorium Fund.

81 Natanael. Doc. no. 1126, tin CO9606, Year 1935. APESP, Pinel Sanatorium Fund.

82 Natanael. Doc. no. 1126, tin CO9606, Year 1935. APESP, Pinel Sanatorium Fund.

83 Natanael. Doc. no. 1126, tin CO9606, Year 1935. APESP, Pinel Sanatorium Fund.

84 Natanael. Doc. no. 1126, tin CO9606, Year 1935. APESP, Pinel Sanatorium Fund.

85 Natanael. Doc. no. 1126, tin CO9606, Year 1935. APESP, Pinel Sanatorium Fund.

86 Natanael. Doc. no. 1126, tin CO9606, Year 1935. APESP, Pinel Sanatorium Fund.

87 Natanael. Doc. no. 1126, tin CO9606, Year 1935. APESP, Pinel Sanatorium Fund.

88 Natanael. Doc. no. 1126, tin CO9606, Year 1935. APESP, Pinel Sanatorium Fund.

89 Natanael. Doc. no. 1126, tin CO9606, Year 1935. APESP, Pinel Sanatorium Fund.

90 Natanael. Doc. no. 1126, tin CO9606, Year 1935. APESP, Pinel Sanatorium Fund.

91

Ruy Laurenti , ‘Homosexuality and the International Classification Of Diseases’, Revista de Saúde Pública, 18 (1984), 344–76531637 10.1590/s0034-89101984000500002, online: https://www.scielosp.org/pdf/rsp/1984.v18n5/344-347/en, accessed 4 March 2024. According to the author, the classification established in the 6th revision was maintained in the 7th (1955). In the 8th revision (1965), ‘homosexuality was removed from the “Pathological Personality” category and placed in the “Sexual Deviations and Disorders” category (code 302), with the specific subcategory becoming 302) – Homosexuality’. The 9th Revision (1975) kept homosexuality in the same category and subcategory, but considering ‘divergent opinions of psychiatric schools, placed the following guideline under the code “Code homosexuality here whether or not it is considered a mental disorder”’.

92 Antonio Carlos Pacheco e Silva. *Sexología y Psiquiatría Transcultural.* Collection of the FMUSP Historical Museum (mimeo), 1971 *apud* Tarelow, Antonio Carlos Pacheco e Silva, *op. cit.* (note 10), 271.

93 Garcia-Diaz, *op. cit.* (note 36), 3; Rosón and Medina Doménech, *op. cit.* (note 25).

94 Rosón and Medina Doménech, *op. cit.* (note 25), 411–412.

95

Michele Zimbalist Rosaldo , ‘Em Direção a Uma Antropologia Do Self e do Sentimento’, RBSE – Revista Brasileira de Sociologia da Emoção, 18, 54, (2019), 39 .

96 Helena. Doc. no. 05, tin CO9576, Year 1929. APESP, Pinel Sanatorium Fund.

97 Marília. Doc. no. 2453, tin CO9642, Year 1939. APESP, Pinel Sanatorium Fund.

